# A brief review of non-invasive brain imaging technologies and the near-infrared optical bioimaging

**DOI:** 10.1186/s42649-021-00058-7

**Published:** 2021-06-25

**Authors:** Beomsue Kim, Hongmin Kim, Songhui Kim, Young-ran Hwang

**Affiliations:** Neural Circuit Research Group, Korea Brain Research Institute (KBRI), 61, Cheomdan-ro, Dong-gu, Daegu, 41068 South Korea

**Keywords:** Imaging techniques, NIR, Organic dyes, CNS

## Abstract

Brain disorders seriously affect life quality. Therefore, non-invasive neuroimaging has received attention to monitoring and early diagnosing neural disorders to prevent their progress to a severe level. This short review briefly describes the current MRI and PET/CT techniques developed for non-invasive neuroimaging and the future direction of optical imaging techniques to achieve higher resolution and specificity using the second near-infrared (NIR-II) region of wavelength with organic molecules.

## Introduction

The human brain controls all the intentional/unintentional movement of the body and complex mental function for high-level living. Various brain defects, such as senile brain diseases and social brain diseases, have gradually increased in recent times with an increase in human lifespan and excessive brain stimulus through environmental changes, social networks/stress, and addictions. However, the prognosis of neurological disorders is generally not favorable despite well-developed neurological treatments. This is primarily because of brain’s characteristic wherein undetectable microdamage may lead to a severe functional defect depending on the damaged area. Therefore, the diagnosis of brain damage generally requires the highest resolution using a noninvasive imaging technique. In the biomedical field, magnetic resonance imaging (MRI) and positron emission tomography (PET)/X-ray computed tomography (CT) are the most popular techniques for brain imaging. Although these techniques are advantageous in terms of rapid and precise imaging of the brain, they are also faced with challenges to represent cellular resolution in a sub-area of the brain. This review provides a brief introduction of brain imaging technologies and their multiple approaches to achieve a breakthrough, especially for the optical imaging technique using the second near-infrared (NIR-II) region of wavelength.

## Main

### MRI

#### Brain MRI and fMRI

**Fig. 1:**
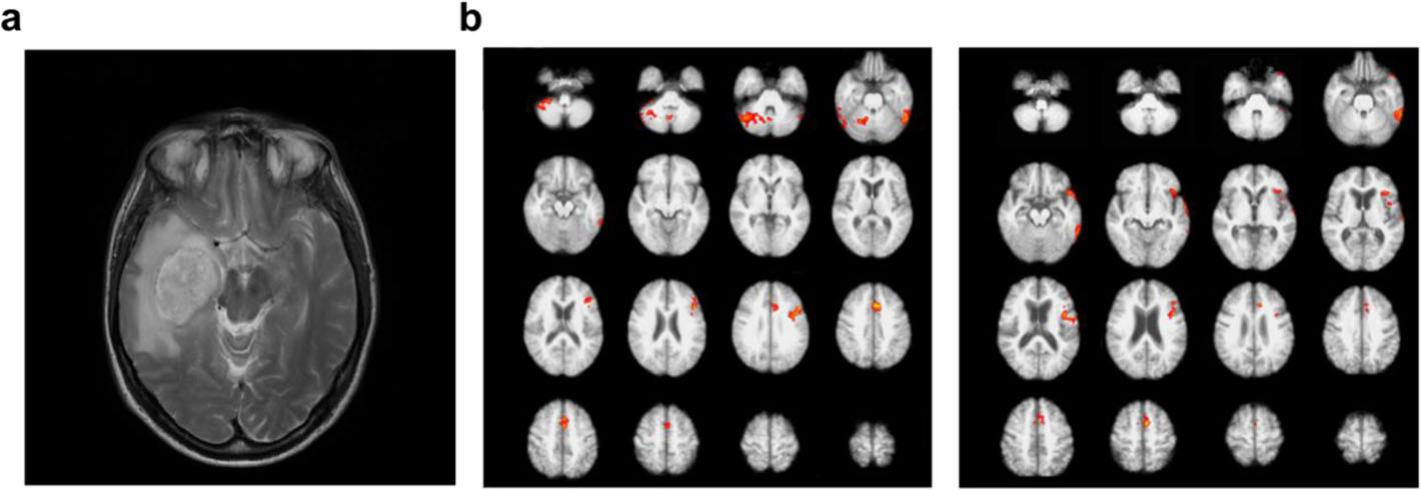
Modality of MRI/fMRI. **a** Axial MRI slice of a patient with a brain tumor. **b** BOLD dependent fMRI scans of verbal fluency task performed by healthy controls (left) and subjects with alzheimer’s disease (right). Adapted with permission from *Crowe et al.* 2017 (**a**) and from *Preti et al.* 2014 (**b**)

MRI uses radiofrequency pulse signals for arraying the hydrogen atom nuclei of the human body alignment. When the opposite resonating hydrogen atom nucleus is returned, the scanner senses the signal and converts it to an image. Therefore, hydrogen-rich soft tissues such as muscle, ligament, blood vessel, and brain are suitable for obtaining MRI images (Fig. 1a).

Because of its noninvasiveness and nontoxic nature, multiple MRI snapshots enable tracking the time-dependent dynamic changes in the human brain. Functional MRI (fMRI) indirectly measures the brain’s neural activity by detecting blood flow changes, relying on the fact that cerebral blood flow occurs simultaneously with nerve activation. The blood-oxygen-level dependent (BOLD) contrast method was developed as fMRI’s primary form in 1990 using paramagnetic deoxyhemoglobin as the natural contrast agent (Ogawa et al. 1990). Since then, fMRI has been widely used for functional brain mapping under various cognitive conditions or neural disorders with millimeter precision through its high spatiotemporal resolution (Sereno et al. 1995; Filippi et al. 2018; Thibault et al. 2018; Chen 2019) (Fig. 1b). The technique is now applied to identify the target region of brain glioma (Ghinda et al. 2018) and epilepsy before surgery (Duncan et al. 2016) and to diagnose several neural disorders (schizophrenia, autism spectrum disorder, mild cognitive impairments, Alzheimer’s disease, bipolar disorder, and major depressive disorder) by comparing patient’s fMRI data with a massive reference dataset of healthy human brains, such as DiaNet and NeuroMark (Eslami et al. 2019; Du et al. 2020). However, the analytical time for extracting signal from noise is the bottleneck to a particular condition such as an emergency. Diffusion-weighted MRI (DWI) is an MRI method to detect the degree of water diffusion. Because of its short time requirement for visualization, DWI is used in the clinic to detect acute cerebral infarction by generating high DWI signals within a few minutes (Boonrod et al. 2018). The visualization of white matter, mainly formed by myelination, is an excellent target for clinical brain MRI, particularly in diagnosing multiple sclerosis (MS) and other neurodegenerative diseases (Inglese and Petracca 2013). However, their specificity cannot be increased to identify a specific biomolecule because the conventional MRI techniques generate imaging contrast based on their different resonance activity of hydrogen atoms (Dregely et al. 2018). The MRI lesion measurements generally show weak correlations with clinical conditions measured on the Disability Status Scale (Neema et al. 2007; Heath et al. 2018).

#### Current contrast agents for MRI

Magnetic materials have been studied in MRI to obtain a better image by increasing the relaxation rate of water molecules and the specificity through the property of the materials (Xiao et al. 2016). Clinical practice preferably uses T1 compared to T2 contrast agents, which can darken the desired area, resulting in less contrast effect and signal distortion. Gadolinium-based T1 contrast agents have advantages based on their high magnetic moment and stability (no electrons bounded), leading to their most extensive use during past decades. Alternatively, manganese and barium are also used as T1 contrast agents. However, those contrast agents have hardly been tried for brain imaging mainly because of their toxicity (Wahsner et al. 2019; Ding et al. 2020).

**Fig. 2:**
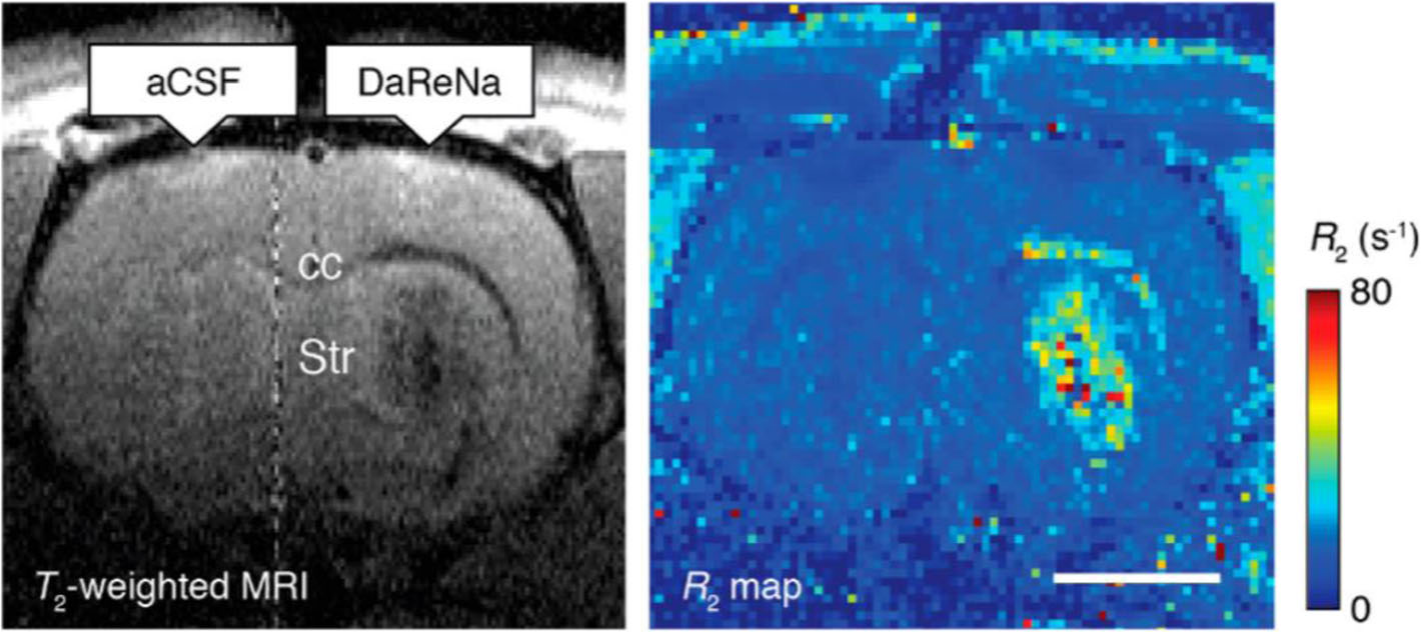
MRI contrast agent. Infusion of the DaReNa, a dopamine sensor (BM3h) coated SPION complex, show the enhancement of contrast and relaxation near the injection site (striatum, Str) and along the corpus callosum (cc). Scale bar = 2 mm. Reprinted with permission from *Hsieh et al.* 2019*. Copyright 2017 American Chemical Society*

Iron is an essential substance for biomaterials such as hemoglobin and can quickly metabolize in the body, making it more biocompatible. However, a ferrous ion (Fe^2+^) needs to transform into a suitable ligand form in order to be used as a T1 contrast medium (Zhou et al. 2019). Initial trials use Fe + salts (e.g., Fe + chloride) and tri−/di-ethylene glycol to synthesize hydrophilic microscopic Fe + oxide nanoparticles, but it generates heterogeneous products causing abnormal MR relaxation times (Li et al. 2012). Superparamagnetic iron oxide nanoparticles (SPION) are then synthesized for multimode imaging because of their nontoxic nature and flexible design, like the dextran-conjugated SPION for monitoring cancer progress through in vivo macrophage MRI (Schleich et al. 2015; Sharkey et al. 2017). In a recent study, SPION conjugated with a dopamine sensor could visualize dopamine in brain MRI in vivo at the animal level (Hsieh et al. 2019) (Fig. 2). Moreover, the ultra-small size of SPIONs (USPIO) conjugated with an Aβ antibody has been developed to diagnose AD (Yang et al. 2011; Luo et al. 2020).

Adapting paramagnetic metalloproteins is a unique approach to generate a ferrous ion-based contrast agent, especially for detecting dopamine in brain MRI. Similar to hemoglobin in BOLD fMRI, the bacterial cytochrome P450-BM3 heme domain (BM3h) also carries a single iron (III) atom, generating different MRI contrast depending on their oxidation state (Shapiro et al. 2010; Brustad et al. 2012). An important finding was that dopamine had an affinity to bind to the substrate-binding pocket of BM3h, thereby causing a decrease in relaxivity, meaning that the enabling dopamine MRI (Brustad et al. 2012; Hsieh et al. 2019) (Fig. 2). Various mutants, including BM3h-8C8, BM3h-9D7, and BM3h-B7, have been identified through protein engineering of multiple rounds of mutagenesis by focusing on its high affinity to dopamine (Angelovski and Tóth 2017). A recent study revealed distinct neuromodulatory actions of striatal dopamine by combining fMRI and molecular imaging techniques for dopamine (Li and Jasanoff 2020). Despite its potential to image specific biomolecules, further studies on MRI are needed for a breakthrough in delivering the modified peptide for human brain MRI (Duro-Castano et al. 2020).

### PET/CT

PET is a noninvasive imaging technique to visualize the dynamic distribution of a radioactive substance (e.g., Fluorine-18 labeled) in the body by detecting its emitted positrons. It can simultaneously take PET and CT images in one machine to show the anatomical localization of PET signal by CT (PET/CT). By using the high signal-to-noise ratio and the penetration efficiency of radioactive molecules, PET can detect the target at the maximum resolution regardless of the cellular composition of the surrounding tissues, unlike other noninvasive imaging techniques such as CT or MRI. Hence, the chance to develop a unique PET probe is relatively flexible, meaning that if a fast synthetic scheme for the PET labeling is available, such as fluorine-18 labeling, a well-developed compound can be tried for PET imaging.

**Fig. 3:**
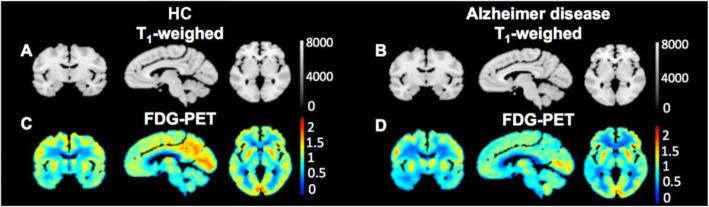
Modality of PET/CT. The standardized glucose uptake value ratios (SUVR) relative to the cerebellum from 18F-FDG PET scans of a healthy control (HC) and an Alzheimer’s disease patient. Adapted with permission from *Kazemifar et al.* 2017

Fluorodeoxyglucose (18F-FDG), the most common PET probe, visualizes glucose metabolism, where its bright signal appears in high-energy demand areas of the body, such as cancers and active neurons. Therefore, 18F-FDG has been used for diagnosing several neurological diseases, including AD, PD, and glioma (Meyer et al. 2017; Kazemifar et al. 2017; Chételat et al. 2020; Quartuccio et al. 2020) (Fig. 3a). Besides 18F-FDG, tracers targeting general phenomena of neurological diseases such as high inflammatory responses have been studied for diagnosing neurodegenerative diseases. Imaging of neuroinflammation has been attempted with tracers of TSPO (18 kDa translocator protein/peripheral benzodiazepine receptor) protein (Kreisl et al. 2013). Although its representative inflammation biomarker is a common feature, the results with various TSPO tracers such as PK-11195, DAA1097, DAA1106, PBR06, PBR28, PBR111, and DPA713 in brain imaging are still controversial to reveal the use of TSPO for differentiating patients from healthy control; this might be due to the expression of TSPO in astrocytes, endothelial cells, and vascular smooth muscle cells (Perrone et al. 2016; Alam et al. 2017; Gui et al. 2020; Lee et al. 2020; Pannell et al. 2020).

**Fig. 4:**
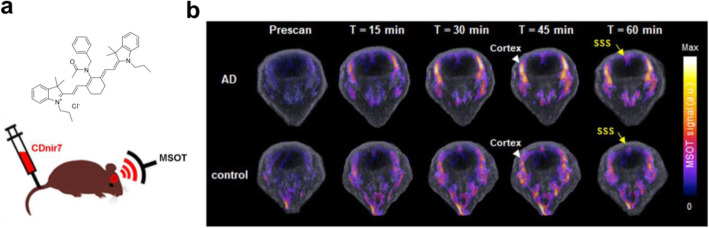
Modality of MSOT imaging. **a** The chemical structure of CDnir7 and the experimental scheme. **b** MSOT signals in the cortex of both AD and control brains before (Prescan) and after the intravenous injection of CDnir7. SSS, superior sagittal sinus. Adapted with permission *Park et al.* 2019a*,* b

Like neuroinflammation, it is often required to develop a novel method if no other promising PET imaging target existed. Forward phenotypic screening can be a choice to suggest a new approach when the appropriate target is not identified yet (Yun et al. 2014). A forward chemical screening using a large number of small fluorescent molecule libraries identified a novel small fluorescent molecule, named CDnir7 (compound of designation near-infrared 7), as the fluorescent probe for selectively labeling activated microglia/macrophage compared to non-activated control (Kang et al. 2014). Interestingly, CDnir7 showed specific labeling of mouse AD brain in vivo using multispectral optoacoustic tomography, meaning that the target of CDnir7 can be used as a novel biomarker for AD bioimaging (Park et al. 2019a) (Fig. 4). Although the target biomolecule for CDnir7 has not been identified yet, its potential to diagnose a neurological disease will lead to further development of CDnir7 as a PET probe similar to a recent dual-modal probe be used either for fluorescence and PET imaging (Kang et al. 2020).

### Near-infrared-II bioimaging

#### Fluorescence imaging

Fluorescence imaging is one of the most commonly used imaging tools in research and clinical settings, applied to either fixed and live specimens (Zhao et al. 2018). The advantages of fluorescence include its highest sensitivity, specificity, and spatial resolution that can reach subcellular levels. However, specific targeting of the desired biomolecule using fluorescence could not be applied for a long time because of the lack of labeling technology. Since the first report of 1941, immunofluorescence labeling techniques have used for tracking a specific biomolecule with the antibody bound with a bright and photostable fluorescent small molecules (Packer 2021). The trackable biomolecules include endogenous proteins, peptides, nucleic acids, glycoproteins, lipids, and self-labeling protein tags that can be recognized by an antibody. The utilization of green fluorescent protein (GFP) in the early 1990s led to numerous studies using a GFP with various purposes in basic research, such as fluorescence tagging of a target protein and fluorescence labeling of a specific cell type of a live organism (Tsien 1998; Ni et al. 2018). However, fluorescence bioimaging based on GFP expression requires transferring a large GFP expression cassette, which is typically achieved by injecting viral particles into the site of interest or generation of a transgenic animal. As both these methods are unsuitable for diagnostic purposes, especially for humans, there was a demand to develop a safe methodology with specificity to a biomolecule and high fluorescence.

Several small organic compounds have recently been reported with characteristics of fluorescence and biomolecule specificity, i.e., CDr20 (compound of cell designation red 20) to detect UDP-glucuronosyltransferase 1a7c enzyme activity, CDg16 (compound of cell designation green 16) for solute carrier 18b1, and TiY (tumor-initiating cell probe yellow) for vimentin (Lee et al. 2018; Kim et al. 2019a; Park et al. 2019b). This approach has advantages of brain imaging, in that the small size of organic compounds does not lead to any genetic modification or genetic material transfer. It may enter the brain’s parenchymal area across the blood-brain barrier (BBB) without additional modification of the compound when injected into the peripheral bloodstream depending on its chemical properties (Miao et al. 2019). Nonetheless, the limited penetration depth of excitation light into the brain is a drawback of fluorescence biomolecules for noninvasive imaging (Hong et al. 2014). Moreover, although it seems to have no apparent toxicity or disturbance for the targeted organ’s in vivo imaging, more detailed biocompatibility and toxicity test need to be performed for the above materials.

#### NIR fluorescence imaging

**Fig. 5:**
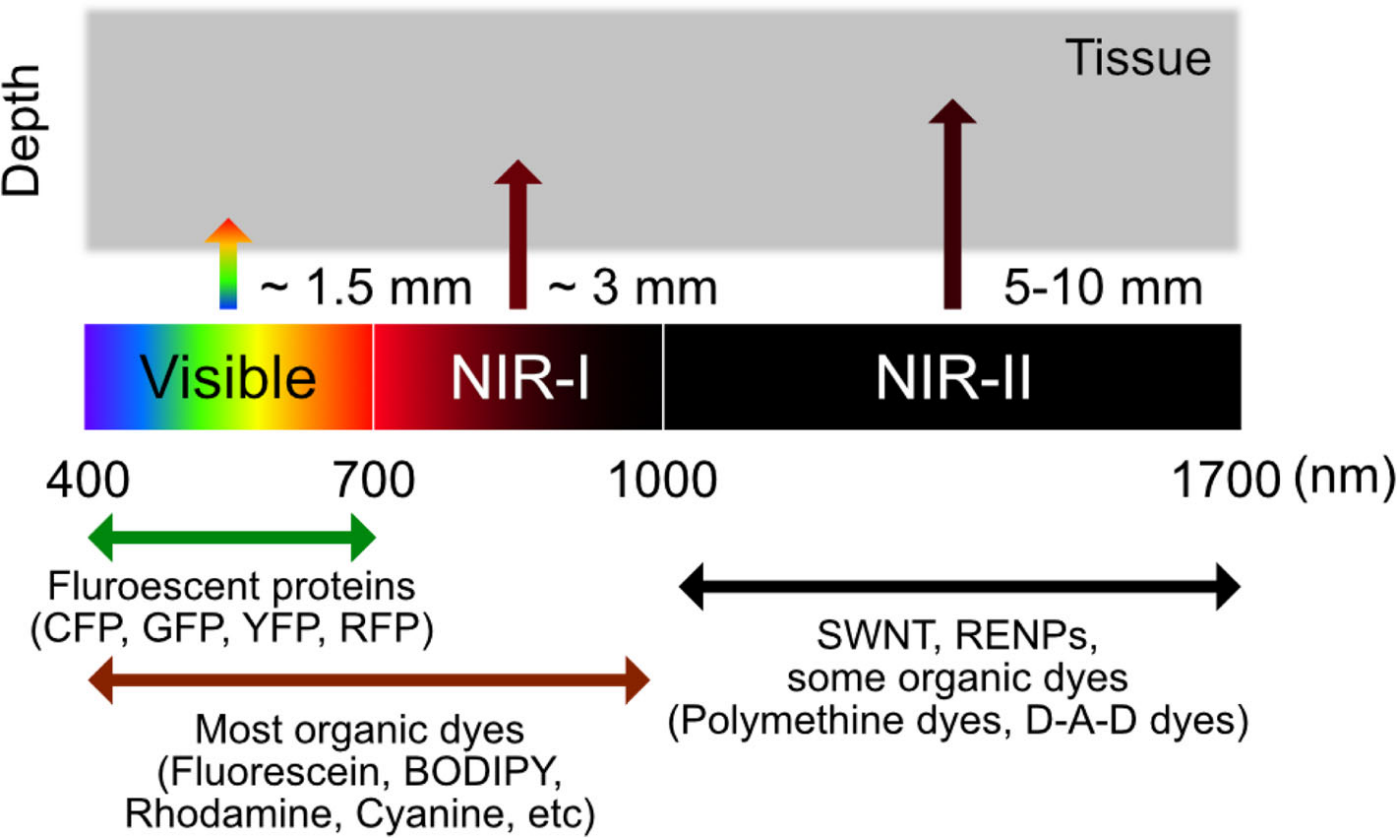
Commonly used materials for fluorescence imaging. Diagrams of the spectral ranges of the major fluorescence materials and its tissue penetration capacity. SWNTs, single walled carbon nanotubes; RENPs, rare-earth doped nanoparticles; D-A-D, donor-acceptor-donor

Biomedical fluorescence imaging has commonly focused on the visible light wavelength (380–700 nm) as its standard practice. For example, FDA-approved hexaminolevulinate emits fluorescence at 635 nm wavelength by producing endogenous protoporphyrin IX, which can be used to visualize nonmuscular invasive papillary bladder cancer (Witjes and Douglass 2007). However, compared to its clear description of the vascular system, other internal organs, including the brain, are difficult to detect because the tissue penetration efficiency of the light was insufficient (Cao et al. 2020). Moreover, tissue barriers in the target internal organs such as skin, fat, and bone can interfere the noninvasive imaging by its intense background noise in visible light. Based on the physical properties of tissue barriers related to wavelength, near-infrared (NIR) light (> 700 nm) has been used to overcome the tissue penetration depth, especially in noninvasive in vivo fluorescence imaging (Luo et al. 2011; Zhang et al. 2016; Wan et al. 2018). Notably, compared to a shorter wavelength of NIR window (700–900 nm, NIR-I), the longer wavelength (1000–1700 nm), called NIR-II, has advantages for deeper penetration because of the low photon absorption rate of barrier tissues and light scattering, and higher spatial resolution for in vivo imaging (Cao et al. 2020) (Fig. 5).

Brain imaging using NIR-II light has been developed in two directions: functional near-infrared spectroscopy (fNIRS) and bioimaging with exogenous NIR molecules. fNIRS measures brain activities noninvasively by measuring oxygenated and deoxygenated hemoglobin like BOLD fMRI (Steinbrink et al. 2006). The advantage of fNIRS is its safety, inexpensiveness, and simplicity compared to fMRI. These advantages led to its rapid development, including the multi-directional fNIRS and probes’ increment for high-density measurement (Shin et al. 2017; Shimokawa et al. 2019; Chiarelli et al. 2020). As cortices can be an appropriate target for fNIRS bioimaging, the current sensitivity has reached to facilitate to detect of the symptoms of neurological disorders, including schizophrenia, stroke, epilepsy, major depressive disorder, AD, and PD (Koike et al. 2013; Hatakeyama et al. 2017; Li et al. 2018; Sirpal et al. 2019; Stuart et al. 2019; Tian et al. 2019; Yang et al. 2019; Ho et al. 2020).

#### Organic molecules used for the near-infrared imaging of brain

**Fig. 6:**
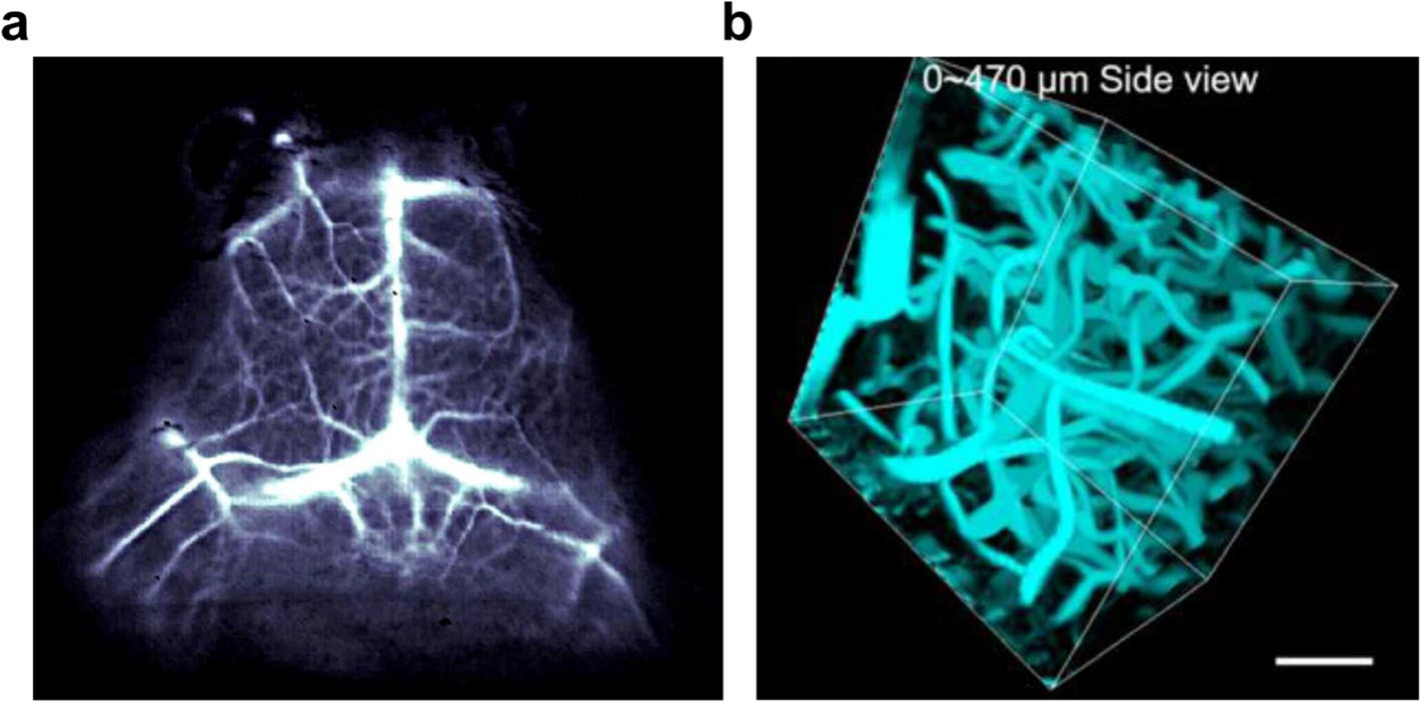
Modality of NIR-IIb fluorescent optical imaging. **a** In vivo mouse brain vessel imaging using a cerium-doped rare earth nanoparticle. **b** 3D reconstruction of cerebral blood vessels of the rhesus macaque based on ICG-mediated NIR-II fluorescence confocal microscopic in vivo imaging up to depth of 470 μm. Adapted with permission from *Zhong et al.* 2017 (**a**) and from *Cai et al.* 2020 (**b**)

On the other hand, exogenous NIR molecules have gradually developed for visualizing brain imaging starting from brain tumors and cerebrovascular disorders. For example, through the intrinsic photoluminescence of single-walled carbon nanotubes (SWNTs) with 1300–1400 nm NIR-II window, it was possible to image blood flow of the mouse brain at a depth of > 2 mm with < 10 μm resolution with ~ 5.3 frames per second (Hong et al. 2014). A cerium-doped rare-earth nanoparticle emits 1550 nm wavelength, also enables in vivo blood imaging of mouse brain with a short exposure time (20 ms) at 980 nm excitation (Zhong et al. 2017) (Fig. 6a). However, it needs to be careful to forward the molecules into the clinic because the SWNTs and the rare-earth doped nanoparticles (RENPs) often show toxicity in vivo depending on their morphology, property, and purity (Huang and Lovell 2017). Besides carbon nanotubes and nanoparticles, NIR range organic compounds also showed brain imaging for intravascular structure. Indocyanine green (ICG), the FDA-approved imaging agent, could visualize cerebrovascular structures of rhesus macaque monkey at a depth of 470 μm with a high spatial resolution (8 μm) by its red-shifted 806 nm maximal absorbance and ~ 900 nm emission in the serum (Cai et al. 2020) (Fig. 6b). A NIR dots tagging an FDA-approved surfactant, pluronic-F127, on the encapsulated *N,N*-diphenyl-naphthalen-1-amine and benzobisthiadiazole (BBTD) showed emitted light beyond 900 nm with ~ 5 μm spatial resolution at 1065 μm penetration depth and was used to image ischemic brains (Alifu et al. 2018). Therefore, it showed that biocompatible organic compounds were successful for noninvasive imaging of the brain’s intravascular structures. However, those materials were idle to target the brain’s parenchymal area because of its limited BBB penetration efficiency and, more importantly, no specificity to label a type of brain cells.

**Fig. 7:**
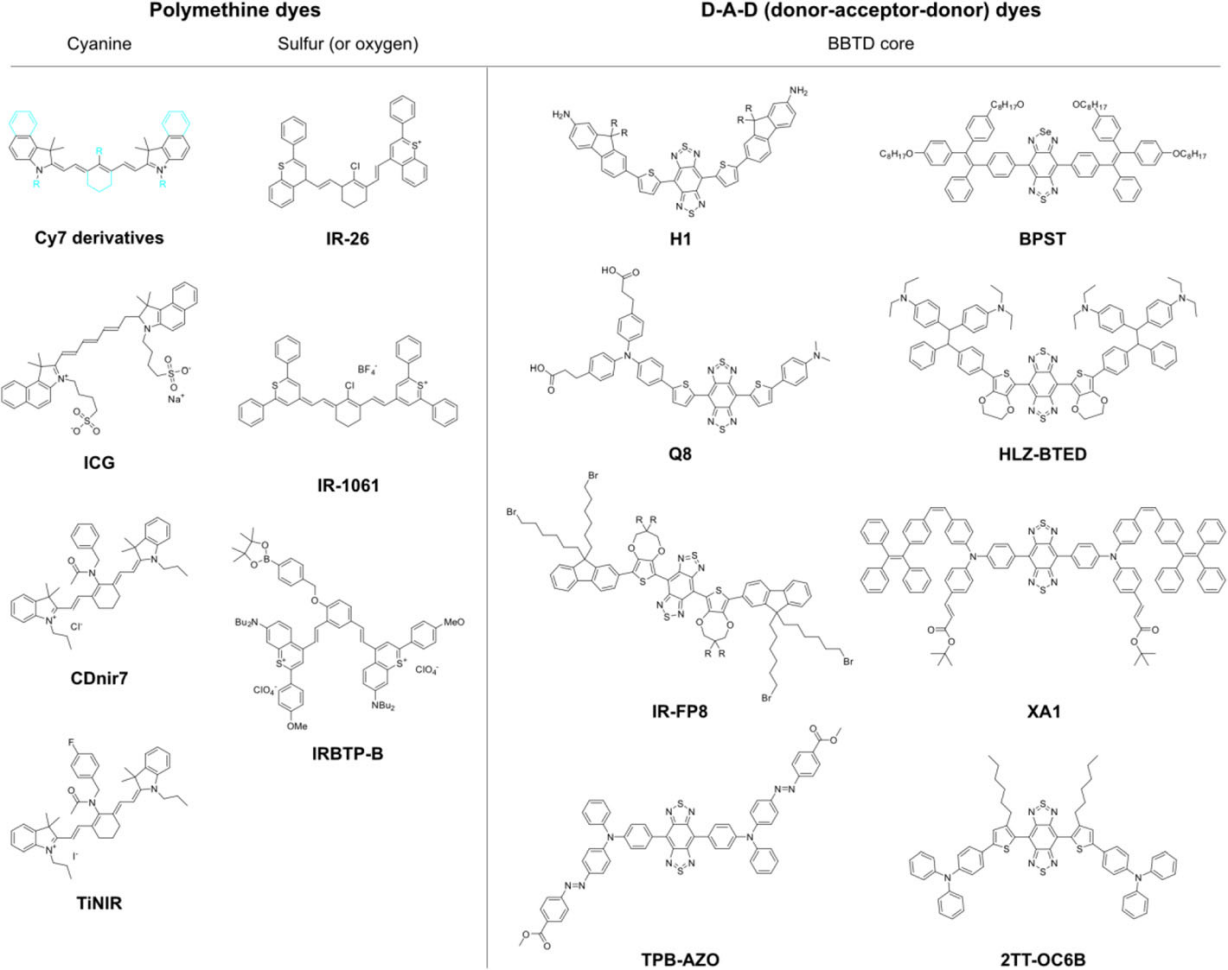
Examples of the chemical structures of NIR organic dyes. Sky colors represent adjustable structures of Cy7 derivatives, such as ICG, CDnir7, TiNIR, etc.; BBTD, benzobisthadiazoles

Over the past decades, researchers have attempted to synthesize several types of NIR fluorescent organic probes having specificity to a biomolecule, which originated from the types of core structures polymethine backbone or donor-acceptor-donor (D-A-D) skeleton, for in vivo imaging (Fig. 7) (Li et al. 2020). The targeted biomolecules were reactive oxygen species, reactive nitrogen species, metal ions, anions, and intracellular pH changes, for achieving high/specific reactivity between the synthetic molecule and the target in live animal cells (Li et al. 2020; Zhao et al. 2021). The biomedical trials resulted in remarkable results for in vivo bioimaging of systemic cancers and hepatotoxicity with NIR-II probes because of its dramatic change of the quantum yield and the spectroscopic properties after reacting with the target biomolecules (Table 1).

**Table 1:** Summary of the NIR organic dyes

Class	Name	Ex.	Emi.	Abs.	Q.Y.	Biomedical Applications	Ref.
Polymethine (Cyanine)	Cy3	550	570	309	0.15	Biotechnologies (labeling, analysis, biomedical imagings)	–
	Cy5	650	670	409	0.27		
	Cy7	743	767	512	0.28		
	ICG	788	830	800	0.04	Clinical angiography, laparoscopic surgery	Kim et al. 2018
	CDnir7	806	821		0.14	Bioimaging – inflammation	Kang et al. 2014
	TiNIR	805	825		0.09	Bioimaging - lung tunor initiating cells	Kim et al. 2019b
Polymethine (Sulfur)	IR-26	785, 808	~ 1050	950	~ 0.05	Bioimaging - Lymphatic systems	Wang et al. 2019
	IR-1061	980	1074	~ 1000	~ 0.02	Bioimaging - Inner organs and blood vessels	Tao et al. 2013
	IRBTP-B	950	~ 1000	905	0.001	Detection - drug-induced hepatotoxicity	Zhao et al. 2021
D-A-D (benzobisthadiazoles)	H1	785	~ 1100	600–1000	0.02	Biomaging - tumor; Imaging-guided surgery	Sun et al. 2017
	Q8	808	1020–1150	600–1000	0.0001	Bioimaging - tumor, Imaging-guided surgery	Qu et al. 2019
	IR-FP8	808	~ 1000	808	0.04	Videorate imaging - hindlimb vessels of mice	Ma et al. 2020
	TPB-AZO		909	690	0.22	Detection - cardiac cyle and heart rate	Zhang et al. 2020
	BPST	715	897	347–711	0.06	Bioimaging - blood, lymphatic vessels, and tumors	Wu et al. 2019
	HLZ-BTED	785	~ 1000	~ 805	0.002	Detection - gastrointestinal function and diseases	Lin et al. 2019
	XA1	808	~ 1000	400–780	0.15	Bioimaging - limb and cerebral vessels	Xu et al. 2020
	2TT-oC6B	808	1014	700	0.11	Bioimaging - brain inflammation	Liu et al. 2020

However, limiting the target biomolecules cause the failure of developed NIR-II probes for penetrating BBB, the most substantial barrier for brain bioimaging, due to the usage of a restricted number of chemical motifs for reactivity to the specific biomolecule (Li et al. 2020). One of the strategies to overcome the limitation is continuously developing novel compounds for the biomolecules. Alternative approache is the targeting thousands of other biomolecules, including thousands of the previously undesignable molecules, such as enzymes/proteins, carbohydrates, and nucleic acids. As mentioned earlier, forward chemical screening based on the diversity of small molecules often generate an unpredictable result that can target other biomolecules, thus showing the brain’s cellular or functional specificity, such as FABP7 binder and CDr3 (Yun et al. 2012).

Interestingly, a recently reported cancer stem cells (CSC) probe, TiNIR (tumor-initiating cell probe near-infrared), was developed by screening 710 cyanine small organic fluorescent compounds. TiNIR showed specificity to lung CSC and was compatible with NIR bioimaging and photoacoustic imaging in vivo (Kim et al. 2019b) (Fig. 7). The subsequent target identification process for TiNIR identified its unexpected target, hemoxygenase-2 (HO-2), for the specific labeling of lung CSC (Kim et al. 2019b) (Table 1). Because there is limited information on HO-2, a constituent form of hemeoxygenase, the forward chemical screening method has proved to be a new path to develop the CSC in vivo bioimaging probe. Future studies to reveal its structure-activity relationship require the discovery of a core structure of TiNIR for binding to the novel target, thus providing fundamental knowledge for the hardly known biomolecule, including its preference for HO-2 posttranslational modifications, which is regulated by the brain condition during seizures, hypoxia, and hypotension (Parfenova and Leffler 2008).

## Future perspective

The brain remains a mystery, but has become a vital organ for diagnosis. There is a high demand to investigate the brain’s functional structures in detail and analyze their responses efficiently; therefore, innovative imaging techniques must be developed to confirm the brain’s fine and functional structure without any physical damage. The current brain imaging techniques such as MRI, fMRI/CT, and PET are reliable and can generate a substantial valuable dataset for brain imaging. Especially, the recent development in the field of medical image analysis called radiomics allows the acquisition of more specified and detailed clinical information from radiological images. Various radiomics models numerate and standardize images based on the features that can distinguish pathological regions from the surrounding structure by the unit of pixels (2D) and voxels (3D). Such conversion of visual information to numeric values enables the quantitative analysis of medical images, which allows discovery of new pathological behavior previously imperceptible. The novel radiomics techniques have been applied to image analysis of brain MRI or CT scans to predict brain metastasis of cancers originated from various organs. (Kniep et al. 2019; Chen et al. 2019).

The application of radiomics, however, is limited to post-data processing of raw image to maximize the amount of information. The original limitation of radiological imaging technology itself, i.e., tissue accessibility and specificity, can only be overcome by the development of a vivid but simple optical imaging device for human in vivo brain imaging. The current findings indicate that NIR-II small molecules are suitable for large animal brain imaging, thus implying their potential applicability to humans.

Although many efforts to develop NIR-II probes have been made to visualize vascular systems or cancer cells, focusing on specific cells of the brain, especially the parenchymal cell types such as neurons and glia, will open new avenues for their use in diagnosing human brain disorders. Novel approaches that can target specific biomarkers of each cell type with desirable optical properties, biocompatibility, and low toxicity will be crucial for noninvasive in vivo human brain imaging.

## The main questions that can be addressed using novel technologies

- Q1. How to measure the level of a neurotransmitter with a non-invasive technique in the live brain?
- Q2. Is it possible to imaging intravascular structure of the live brain in a sub-millimeter resolution?

## Data Availability

The datasets used and/or analyzed during the current study are available from the corresponding author on reasonable request.

## Abbreviations

MRI: Magnetic resonance imaging
PET/CT: Positron emission tomography / X-ray computed tomography
SPECT: Single-photon emission computed tomography
NIR-II: Second near-infrared
fMRI: Functional MRI
BOLD: Blood-oxygen-level dependent
DWI: Diffusion-weighted MRI
MS: Multiple sclerosis
SPION: Superparamagnetic iron oxide nanoparticles
USPIO: Ultra-small size of SPIONs
BM3h: BM3 heme domain
18F-FDG: Fluorodeoxyglucose
TSPO: 18 kDa translocator protein/peripheral benzodiazepine receptor
CDnir7: Compound of designation near-infrared 7
CDr20: Compound of designation red 20
CDg16: Compound of cell designation green 16
TiY: Tumor-initiating cell probe yellow
BBB: Blood-brain barrier
fNIRS: Functional near-infrared spectroscopy
ICG: Indocyanine green
BBTD: Benzobisthiadiazole
CSC: Cancer stem cells
TiNIR: Tumor-initiating cell probe near-infrared
HO-2: Hemoxygenase-2
SWNTs: Single walled carbon nanotubes
RENPs: Rare-earth doped nanoparticles
